# Service Dog Training Interventions for Veterans with Post-Traumatic Stress: Examining Gender-Based Differences in Psychosocial Outcomes

**DOI:** 10.3390/healthcare14091253

**Published:** 2026-05-06

**Authors:** Shahar Almog, Cheryl A. Krause-Parello, Alejandra Quintero, Deborah Taber, Erika Friedmann

**Affiliations:** 1 Division of Research, Canines Providing Assistance to Wounded Warriors (C-P.A.W.W.^®^), Institute for Human Health and Disease Intervention, Florida Atlantic University, Boca Raton, FL 33431, USA; salmog@fau.edu (S.A.); ckrausep@health.fau.edu (C.A.K.-P.); aquintero2014@fau.edu (A.Q.); 2 School of Nursing, University of Maryland, Baltimore, MD 21201, USA; dtaber@umaryland.edu

**Keywords:** service dog training program, human–animal interaction, gender-based differences, canine assistance, female veterans, male veterans, veterans

## Abstract

**Background:** Poor mental health is prevalent among veterans who suffer from post-traumatic stress disorder (PTSD) and other psychiatric conditions. Canine-assisted interventions may improve psychological and social health in veterans. The parent study, a randomized controlled trial, revealed improvements in PTSD following both a service dog training program and an active control condition consisting of virtual dog training lessons. Thus, in the present post hoc secondary analysis, we analyzed both groups together (pooled arms) to examine gender-based differences in the effects of the altruistic service dog training programs on psychosocial outcomes. **Methods**: Veterans (*N* = 59) participated either in hands-on (with a dog and trainer) or virtual (no dog present) dog training programs over eight weeks and completed self-reported psychosocial measures before and after the program. Mixed-effect linear models were used to assess the interaction between time and gender on a series of psychosocial outcomes in the pooled sample. **Results**: The findings supported greater psychosocial improvements for female participants compared to male participants, including significant improvements in PTSD, perceived stress, and perceived physical health, and feeling greater closeness and lower avoidance in close relationships (*p*s < 0.05). The results revealed moderate to large effect sizes among female participants, suggesting meaningful clinical effects of the interventions (*d*s = 0.47–0.70). **Conclusions**: While the secondary analysis and small sample size limit causal inferences, the exploratory evidence suggests greater improvements in psychosocial health in female veterans after participating in the service dog training programs. Future research should tailor interventions to optimize the therapeutic effects for male and female veterans and identify other individual characteristics involved, such as combat exposure or post-traumatic stress symptom severity.

## 1. Introduction

Post-traumatic stress disorder (PTSD) is a psychiatric condition that may develop following experiencing or witnessing a traumatic event. Post-traumatic stress symptoms include hyperarousal, alterations in cognition and emotion, re-experiencing (e.g., flashbacks, nightmares), and behavioral changes (e.g., avoidance), translating into physiological, psychological, and social deficits [1]. With evolving diagnostic criteria for PTSD, it is estimated that up to 30% of United States (US) veterans suffer from post-traumatic stress symptoms [2]. The lifetime prevalence of PTSD in female US veterans is significantly higher than that of their male counterparts [3]. Female veterans are not only at risk for PTSD due to combat exposure but also due to civilian and/or military sexual trauma, leading to more pronounced comorbid psychiatric symptoms [3,4]. Increased rates of comorbid depression and anxiety disorders among veterans with PTSD may also lead to increased overall health risks [5]. With high rates of veterans choosing not to engage in or dropping out of formal psychotherapy practices [6,7], canine assistance has emerged as a complementary option for PTSD symptom management, offering psychological and social benefits [8,9].

Accumulating evidence suggests psychiatric service dogs alleviate post-traumatic stress symptoms and improve psychological and social health in veterans [9,10]. Qualitative evidence also suggests improvements in physical health and relationship building [9]. Per veterans’ reports, a trained service dog may improve their sense of safety, provide companionship, create a calming effect when needed, improve sleep, promote healthy behaviors such as increased physical activity, reduce isolation, and increase social interactions [11,12]. Several studies also investigated the effect of the veteran’s involvement in their own service dog training on PTSD, using different models, including a weekly training program over 14 weeks [13] or up to 12–18 months [14], with the dog living in the veteran’s home, or a 3-week intensive daily training program before taking the service dog home [15,16]. These studies found significant reductions in PTSD symptoms and improvements in quality of life among owner-trainer veterans compared to a waitlist control group [13,14,16]. Using a different model in a small pilot study, veterans participated in six dog training sessions over two weeks, training service dogs for other veterans [17]. Compared to the waitlist control, the training group showed PTSD symptom reduction with moderate to large effect sizes. Still, the research on dog training interventions for veterans is limited and gaps in representation remain; for example, most studies included predominantly White and male samples [18].

Although a minority among veterans, the growing number of female veterans requires the VA health care system to address their unique health needs [19,20]. While research on female veterans and their unique health needs is growing, the call for more clinical research with female veterans and gender-based analysis remains [21]. Relevant to animal-assisted interventions, some literature suggests gender-based differences in the dyadic interaction with dogs. For example, women talk with their dogs more than men [22], women exhibit more positive behaviors and attitudes towards animals and pets [23], and shelter dogs display more stressful behaviors when walked by men compared to women [24]. Together, gender-based differences may play a role in the effectiveness of dog training interventions, yet the scarcity of research involving female veterans or gender-based analysis limits the understanding and the development of targeted interventions.

### The Parent Study

The parent study of the present secondary analysis aimed to assess the effect of an 8-week altruistic intervention of service dog training (for other veterans) on PTSD symptoms and related biomarkers (reported in [25,26]). Participants (47.5% female) were randomized into one of two training groups: hands-on or virtual programs. Participants either participated in service dog training sessions, with a dog and a trainer (i.e., experimental group), or watched virtual service dog training modules, without engaging with dogs (i.e., active control group). Both training programs included 8 weekly one-hour sessions (for more details see [25]). The participants in the virtual training group were notified that upon study completion, they would be able to participate in training sessions with dogs in the training facility. The results showed improvement in PTSD symptoms regardless of group membership (hands-on or virtual). The authors noted that many participants lived with a pet dog, and although not systematically assessed, many participants reported applying what they had learned with their own dogs, possibly gaining indirect benefits. Additionally, upon the study’s completion, many participants in the control group chose to participate in the dog training program at the training facility. It is possible that the virtual program was perceived as a preparatory part of the program, which yielded some psychological benefits. Lastly, midway through the study, after 18 participants completed their participation, the COVID-19 shutdown forced the study to halt. When the study resumed, study procedures were modified to comply with the COVID-19 restrictions, affecting both groups and possibly the outcomes.

During the COVID-19 pandemic, the hands-on intervention was moved outdoors, and rather than one participant per dog, in each session several participants worked with one dog and one trainer, thus having less contact with the dog. The virtual sessions, originally delivered at the same training facility, were moved online to the participants’ homes. Similar to other veteran health care and interventions that were adapted to remote telehealth formats and were well accepted and effective [27,28], the at-home virtual session might have induced a greater sense of safety and benefits. These changes, together with some evidence on the beneficial effects of dog videos on stress and anxiety [29,30], may explain the improvements found in both the hands-on training and virtual training groups, potentially with different underlying mechanisms. Acknowledging the unexpected and limited data to assess the pandemic effects, exploratory analyses revealed no differences between the pre- and post-COVID-19 cohorts, and so we did not control for COVID-19-related factors any further in the present secondary analysis. The two training groups in the parent study did not differ in any of the demographics, military history, pet ownership, or PTSD status at baseline. An exploratory analysis revealed differences between male and female participants in PTSD symptoms over the course of the study (see pgs. 1111–1114 in [25]), leading to the present secondary analysis. As preparatory analyses, we also tested the time by group by gender interaction for the different psychosocial outcomes. The mixed effect models revealed non-significant interactions in all psychosocial outcomes reported herein (see Table S1 in the Supplemental Material).

Therefore, due to the similarities between the hands-on and the virtual training groups in participant characteristics (e.g., a high percentage of dog owners), in the intervention components (e.g., lesson content), and results (i.e., no differences between groups) for the present secondary analysis, we analyzed the groups together for gender as an effect modifier. We aimed to assess the differences between female and male veterans in the response to altruistic service dog training interventions on self-reported psychosocial outcomes in the context of PTSD.

## 2. Materials and Methods

### 2.1. Setting and Participants

The service dog training sessions and study procedures took place at the Warrior Canine Connection (WCC; http://warriorcanineconnection.org/ accessed on 19 March 2026) training facility in a mid-Atlantic state in the United States from September 2019 until March 2023, with a break due to COVID-19 pandemic-related restrictions. The WCC organization trains service dogs for disabled veterans by involving fellow veterans in the long training process. Following the WCC program inclusion and exclusion criteria, the two-arm study aimed to enroll 60 veterans who served in the military on or after 11 September 2001, self-reported ever being diagnosed with PTSD, understood English, and could provide informed consent. While an inclusion criterion of having a current PTSD diagnosis is typical in the literature, PTSD is considered a chronic condition, and residual symptoms may linger even after successful treatment [31,32]. Therefore, the parent study aimed to recruit a more inclusive sample of veterans with ever-diagnosed PTSD and different levels of symptom severity. Veterans were excluded if they reported fear of dogs, allergy to pet dander, active substance use disorder, or history of animal abuse (to ensure canine safety).

### 2.2. Procedures

Participants who met eligibility criteria were randomized into one of the two training programs: a hands-on dog training with a dog and trainer or a virtual dog training, with the option to complete the regular WCC program after study completion. Participants were randomized in blocks of four to balance gender in the two groups. After giving informed consent, all participants completed eight weekly, one-hour-long training sessions over eight weeks, delivered by an experienced service dog trainer. Psychosocial outcomes were collected at Week 1 (i.e., baseline) before the first session, Week 4, and Week 8 after the last session. Participants received a $20 gift card for each data collection session they completed (up to a total of $60). At the last session, they also received a military challenge coin made especially for the study, as gratitude and recognition for their altruistic participation. For the present secondary analysis, we used all available data from baseline and Week 8 from 59 participants who provided at least one measurement. All procedures were approved by the University of Maryland Baltimore Institutional Review Board (IRB) under protocol HP-00083872 and the Institutional Animal Use and Care Committee under protocol #1218021. The present secondary analysis was deemed Not Human Subjects Research by this IRB under protocol HP-00116877.

### 2.3. Interventions

The service dog training program was developed by the WCC organization (accredited by Assistance Dogs International, https://assistancedogsinternational.org, accessed on 10 March 2026). The program trains service dogs for veterans with disabilities by involving other veterans in the different activities during the long training process (e.g., socializing puppies, training the service dogs alongside the professional trainers). This approach enables one service dog to help more than 60 veterans who engage with it as the puppy is raised, trained, and ultimately paired with a veteran. The parent study utilized the 8-session WCC training modules (for more details on the modules’ content see [25]). The virtual lessons for the control group (from https://e-trainingfordogs.com accessed on 9 October 2019) followed the WCC modules and were similar in content.

In general, engaging in altruistic activities is beneficial for well-being [33]. Specifically, the WCC approach provides opportunities for the volunteer-veteran to engage in activities with the dog that can benefit the veteran’s own psychosocial health. For example, by using and practicing positive reinforcement techniques with the dogs, the volunteering trainer-veteran has the opportunity to reinforce their own positive emotions, sense of safety, and trust. The training activities also require the trainer-veterans to focus on the dog in potentially uncomfortable environments, helping the veteran cope with situations that may be challenging (e.g., loud noise). Lastly, the altruistic (i.e., veteran-to-veteran) training program enhances a sense of purpose and provides opportunities to socialize and engage with other members of the community [34].

### 2.4. Measures

#### 2.4.1. Psychosocial Health Outcomes

The severity of post-traumatic stress symptoms was assessed with the 20-item PCL-5 scale [35]. Participants rated the severity of symptoms during the past month from 0 (not at all) to 4 (extremely). Sum scores range from 0 to 80 with higher scores reflecting more severe symptomology. A score of 31–33 suggests a probable PTSD, a reduction of minimum of 5 points may indicate response to treatment, and a reduction of minimum of 10 points may indicate a reliable and meaningful response to treatment [35]. The scale demonstrated good consistency at baseline (*α* = 0.95, *n* = 59) and at Week 8 (*α* = 0.96, *n* = 50). Although exploratory gender-based results from Weeks 1, 4, and 8 were reported elsewhere [25], we repeated the post-traumatic stress symptom analysis (with baseline and Week 8, consistent with all other models in the present report) for a holistic presentation of the participants’ psychosocial health outcomes in the context of PTSD.

Perceived stress was assessed with the Perceived Stress Scale (PSS-10) [36]. The 10-item scale was used to assess the individual’s frequency of feeling capable of coping with stressful events in their lives during the past month. Statements were rated from 1 (never) to 5 (very often). Sum scores range from 10 to 50, with higher scores reflecting greater perceived stress. The scale demonstrated good consistency at baseline (*α* = 0.87, *n* = 59) and at Week 8 (*α* = 0.92, *n* = 51).

Anxiety was assessed with the Patient-Reported Outcome Measurement Information System (PROMIS) Anxiety Short Form 8a [37,38]. The 8-item scale assesses frequency of fear and anxiety suffering over the past seven days on a scale from 1 (never) to 5 (always). The raw sum scores range from 8 to 40 with higher scores reflecting more severe anxiety. The scale demonstrated good consistency at baseline (*α* = 0.93, *n* = 58) and at Week 8 (*α* = 0.96, *n* = 51).

Depression was assessed with the PROMIS Depression Short Form 8a [37,38]. The 8-item questionnaire assesses the frequency of depressive symptoms over the past seven days on a scale of 1 (never) to 5 (always). The raw sum scores range from 8 to 40 with higher scores reflecting more severe depression. The scale demonstrated good consistency at baseline (*α* = 0.95, *n* = 59) and at Week 8 (*α* = 0.97, *n* = 51).

Positive affect was assessed with the PROMIS Positive Affect-Short Form 15 (PA 15a) [39]. The 15-item scale assesses feelings that reflect pleasurable engagement with the environment (e.g., happiness, joy, excitement) over the past seven days, on a scale from 1 (not at all) to 5 (very much). The raw sum scores range from 15 to 75, with higher scores reflecting greater positive affect. The scale demonstrated good consistency at baseline (*α* = 0.95, *n* = 59) and at Week 8 (*α* = 0.97, *n* = 51).

Resilience (i.e., the ability to adapt and deal with challenging events) was assessed with the 10-item Connor–Davidson Resilience Scale 10 [40]. Participants rate 10 statements (e.g., “I am able to adapt when changes occur”) from 0 (not true at all) to 4 (true nearly all the time) based on the past month. Sum scores range from 0 to 40, with higher scores reflecting greater resilience. The scale demonstrated good consistency at baseline (*α* = 0.88, *n* = 58) and at Week 8 (*α* = 0.94, *n* = 51).

Perceived satisfaction with participation in social activities was assessed with the Satisfaction with Participation in Discretionary Social Activities Short Form 7a (PROMIS SPDSA) [41]. The 7-item scale assesses contentment with friends and leisure activities over the past seven days. The seven items (e.g., “I am satisfied with my ability to do leisure activities”) are assessed on a scale from 1 (not at all) to 5 (very much). The raw sum scores range from 7 to 35, with higher scores reflecting greater satisfaction with participation in social activities. The scale demonstrated good consistency at baseline (*α* = 0.95, *n* = 59) and at Week 8 (*α* = 0.95, *n* = 51).

Quality of close relationships was assessed with the Relationship Scale Questionnaire [42,43]. In the 30-item scale, participants rate their feelings (e.g., “I worry about being alone”) on a scale from 1 (not at all like me) to 5 (very much like me). The scale has two subscales, representing two dimensions related to attachment style and behaviors. Scores reflect the participant’s place on a continuum from closeness to avoidance (or independence) in their close relationships, and from feeling security to anxiety in their relationships. Scores range from 18 to 90 on the close/avoidant subscale, and from 16 to 80 on the secure/anxious subscale. Higher scores reflect greater feelings of avoidance and anxiety in relationships. The subscales demonstrated good consistency at baseline (*α* = 0.85, *n* = 59; *α* = 0.85, *n* = 59) and at Week 8 (*α* = 0.84, *n* = 51; *α* = 0.88, *n* = 51), for close/avoidance and secure/anxiety subscales, respectively.

Perceived satisfaction with relationships and activities with friends and family was assessed with the Satisfaction with Social Roles and Activities (SSRA) Short Form 4a (PROMIS-SSRA) [41]. Four items (e.g., “I am satisfied with my ability to do things for my family”) are assessed on a scale from 1 (not at all) to 5 (very much). The raw sum scores range from 4 to 20, with higher scores reflecting greater satisfaction with social roles and activities. The scale demonstrated good consistency at baseline (*α* = 0.82, *n* = 59) and at Week 8 (*α* = 0.92, *n* = 51).

Perceived companionship or the availability of someone with whom to share enjoyable social activities was assessed with the Companionship Short Form 4a (PROMIS-C) [44]. Four items (e.g., “Do you have someone with whom to have fun?”) are assessed on a scale from 1 (never) to 5 (always). The raw sum scores range from 4 to 20, with higher scores reflecting greater perceived available companionship. The scale demonstrated good consistency at baseline (*α* = 0.93, *n* = 58) and at Week 8 (*α* = 0.95, *n* = 51).

Perceived quality of life was assessed with the Veterans RAND 12-Item Healthy Survey (VR-12) [45]. The VR-12 was developed from the Veterans RAND 36 Item Health Survey, which was developed from the MOS RAND SF-36, Version 1.0. The SF36^®^ and SF-12^®^ are registered trademarks of the Medical Outcomes Trust. The 12 items are summarized into two scores: perceived physical health, with possible scores ranging from 6 to 26, and perceived mental health with possible scores ranging from 6 to 33. Higher scores reflect better perceived physical health or better perceived mental health. The subscales demonstrated good consistency at baseline (*α* = 0.86, *n* = 59; *α* = 0.85, *n* = 58) and at Week 8 (*α* = 0.85, *n* = 51; *α* = 0.89, *n* = 51) for perceived physical health and perceived mental health, respectively.

Suicidal ideation was assessed with the Beck Scale for Suicide Ideation (BSS) [46,47]. The 21-item scale identifies the presence and severity of suicidal ideation while assessing suicidal plans, deterrents to suicide, and the level of openness to revealing suicidal thoughts. In the current analysis, due to survey errors, we used only the sum of the screening items (items 4 and 5: desire to kill oneself, action in life-threatening situation) to determine suicidality at baseline. A score of 0 reflects no suicidality. Any score other than 0 reflects some suicidality. The percentage of participants exhibiting suicidality was used to describe the sample and gender-based groups.

#### 2.4.2. Participant Characteristics

Participant demographics and characteristics, including age, gender, race, ethnicity, highest education level, marital status, living arrangements, military history, and pet ownership were collected at baseline.

### 2.5. Analytic Plan

Descriptive statistics were calculated for sample demographics and characteristics. To assess the effect of the dog training interventions on each psychosocial outcome from baseline (pre-intervention) to Week 8 (post-intervention), and whether the effect varied between females and males, we ran multiple linear mixed-effects models (LMMs). To assess the interaction between time and gender, the models included the fixed effects of time (as categorical Baseline/Week 8), which captures the effects of the interventions, gender (female/male), the interaction between time and gender, and random (participant) intercepts, with variance components covariance. We used the maximum likelihood method of estimation with all available data, as LMM handles random missing data well [48]. Significance of post hoc comparisons was Bonferroni-adjusted. Overall, *p* values should be interpreted with caution due to multiple analyses of correlated variables. Thus, regardless of significance, parametric paired *t*-tests were conducted to generate within-group effect sizes (Cohen’s *d*) from baseline to Week 8 in female and male participants separately. Cohen’s *d* values (calculated from the standard deviation of change scores) of 0.2, 0.5, and 0.8 were interpreted as cutoffs for small, moderate, and large effects, respectively, with moderate effect sizes corresponding to meaningful change perceived by patients [49]. Compatible with a medium sample size, normality of distribution was assumed if the absolute z-skewness (i.e., skewness/SE skewness) or z-kurtosis (i.e., kurtosis/SE kurtosis) was less than 3.29 [50]. SPSS 31.0.0.0 (IBM Corp., Armonk, NY, USA) was used for all analyses, and Sigma Plot 14.50 (Systat Software, Inc., San Jose, CA, USA) was used for generating the graphs.

## 3. Results

### 3.1. Participants

Of the 60 participants who consented, one participant withdrew before providing any data. Thus, the analyzed sample included 59 participants who provided data in at least one measurement time. Missing data at Week 8 were from eight participants who dropped out of the study at different stages: five female (three from the in-person training group) and three male participants (one from the in-person training group). Reasons for attrition included illness, traveling, death in the family, lack of time, or inconvenience (see [25]). The eight participants did not differ in mean PTSD score at baseline from participants who provided full data (*M*[*SD*] = 42.38 [14.25] and 41.18 [17.88], respectively, *t*_(57)_ = −0.18, *p* = 0.858). Table 1 presents the sample characteristics for the full sample and for female and male participants separately. The full sample (mean age of 47.37, *SD* = 11.38) was mostly White (62.7%), non-Hispanic (88.1%), and educated, with 67.8% reporting a bachelor’s degree or higher. Overall, female and male participants did not differ in age, race, ethnicity, education, marital status, pet ownership, post-traumatic stress severity, or percentage of participants reporting some level of suicidality at baseline (*p*s > 0.181). A significantly higher percentage of male participants reported combat exposure than female participants (71.0% and 35.7%, respectively, *p* = 0.009).

### 3.2. Psychosocial Outcomes

The means and standard deviations of the psychosocial outcomes at baseline (pre-intervention) and at Week 8 (post-intervention) for the full sample and for female and male participants separately are presented in Table 2. On average, PTSD scores in female participants were reduced by more than 11 points, suggesting meaningful improvements in symptoms.

**Figure 1:**
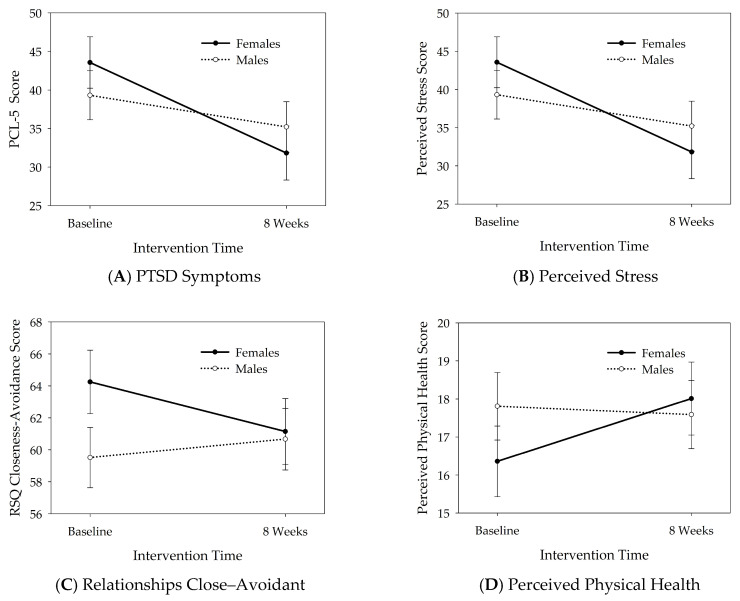
Psychosocial results: time (pre–post) by gender (female–male) interaction. Significant changes in female compared to male participants following service dog training interventions: reduced post-traumatic stress disorder (PTSD) symptom severity (**A**), reduced perceived stress (**B**), reduced avoidance and increased closeness in close relationships (**C**), and improved perceived physical health (**D**). The circles and bars represent the estimated marginal means and standard error of the means. PTSD results were also shown in exploratory analysis in the parent study [25].

Table 3 presents the results of the linear mixed-effects models assessing whether female and male participants were affected differently by the training interventions (i.e., the interaction between time and gender), and the associated within-subject effect sizes with 95% confidence intervals for female and male participants separately. The mixed effects modeling analyses revealed a significant interaction between time and gender for PTSD symptoms (*F*_1,53.06_ = 4.33, *p* = 0.042), perceived stress (*F*_1,54.57_ = 6.26, *p* = 0.015), relationships quality closeness–avoidance (*F*_1,52.37_ = 5.46, *p* = 0.023), and perceived physical health (*F*_1,52.17_ = 5.58, *p* = 0.022). The results are depicted in Figure 1. Compared to baseline, at post-intervention, female participants reported significantly reduced PTSD (*p* < 0.001, *d* = 0.70), reduced perceived stress (*p* < 0.001, *d* = 0.67), reduced avoidance in relationships (i.e., increased closeness, *p* = 0.025, *d* = 0.47), and increased (i.e., improved) perceived physical health (*p* = 0.007, *d* = −0.61), with moderate to large effect sizes. Male participants did not show any significant changes from baseline to post-intervention in these outcomes (*p*s > 0.104). The effect sizes in male participants reached small to moderate levels for PTSD symptoms (*d* = 0.42), small to minimal for perceived stress (*d* = 0.14) and perceived physical health (*d* = 0.06), and were in the opposite direction for relationships quality closeness–avoidance (*d* = −0.17).

**Figure 2:**
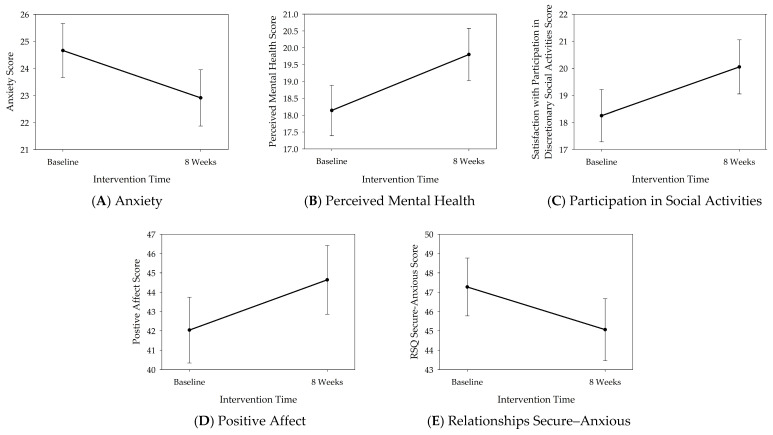
Psychosocial results: the fixed effect of time. Significant improvements in scores over time, with no differences between female and male participants; however, effect sizes were greater in females following service dog training interventions: reduced anxiety (**A**), improved perceived mental health (**B**), increased satisfaction with participation in discretionary social activities (**C**), increased positive affect (**D**), and reduced anxiety and increased security in close relationships (**E**). The circles and bars represent the estimated marginal means and standard errors of the means.

While the interaction between time and gender was not significant in all other outcomes, the fixed effect of time was significant for anxiety (*F*_1,52.66_ = 5.34, *p* = 0.025), satisfaction with participation in discretionary social activities (*F*_1,52.29_ = 8.24, *p* = 0.006), and perceived mental health (*F*_1,53.01_ = 7.47, *p* = 0.008). The fixed effect of time was trending for positive affect (*F*_1,52.90_ = 3.81, *p* = 0.056) and for feeling more secure (than anxious) in relationships (*F*_1,53.02_ = 3.38, *p* = 0.072). These results suggest that improvements were reported by both female and male participants. Still, female participants showed small to moderate effect sizes from baseline to post-intervention (*d*s = 0.36–0.54), which were consistently higher than the effect sizes shown in male participants (*d*s = 0.12–0.24), suggesting that the effect of time was potentially driven by female participants (the results are depicted in Table 3 and Figure 2). No effects were observed in depression, resilience, satisfaction with social roles, and companionship scores.

With clinical relevance and for exploratory and descriptive purposes, we compared the subgroup of participants who reported suicidality at baseline (*n* = 16) with participants who reported no suicidality. Participants who reported suicidality had significantly more severe PTSD (i.e., higher scores) at baseline compared to participants without suicidality (*M*[*SD*] = 55.88 [11.04] and 35.93 [16.15], respectively, *t*_(57)_ = −4.55, *p* < 0.001) but did not differ in PTSD change scores, from pre- to post-intervention (*M*[*SD*] = 8.85 [4.06] and 7.22 [2.26], respectively, Mann–Whitney U = 262.00, *p* = 0.634). Of the 16, five participants (i.e., two females and three males) reported no suicidality at Week 8, nine reported suicidality (no change), and two dropped out of the study. One female and one male who were not suicidal at baseline reported some suicidality at Week 8.

## 4. Discussion

The present secondary analysis aimed to explore whether female and male participants differed in their self-reported psychosocial health outcomes following completion of eight-week service dog training programs. Overall, our findings showed that compared to male participants, female participants reported greater improvement in PTSD symptoms, reduced perceived stress, improved perceived physical health, and improvement in social outcomes (i.e., feeling more closeness and less avoidance in close relationships). The effect sizes in the female participants were moderate to large, potentially clinically meaningful. Although the male participants reported some change in the expected direction, the effect sizes were generally smaller than those observed in the female participants.

While scale score thresholds for clinical improvement are not well established for all scales, effect sizes in the female participants aligned with the observed mean reduction in PTSD (PCL-5) scores of ~11 points, which conventionally reflects a meaningful response to treatment [35]. The observed significant mean reductions of 3–4 points in perceived stress (PSS-10) reflect similar improvements that were seen in another dog training intervention [14] and in a mindfulness intervention in female veterans [51].

Female and male participants also reported improvements in anxiety, satisfaction in participation in discretionary social activities, perceived mental health, and feeling more secure and less anxious in close relationships. Female participants showed improvements, with small to moderate effect sizes in these outcomes, while effect sizes among male participants were minimal to small. Together, our results suggest that dog training programs may be more beneficial for female compared to male participants. However, many limitations and potential confounders exist, and our findings should be cautiously interpreted and used mostly for hypothesis generating.

Our findings, which align with findings of multiple positive psychosocial outcomes following dog training interventions [18], support the call for more female veteran interventional research and gender-specific reporting of results [21]. Specifically, research on canine-assisted interventions for females are lacking [18], as veteran-related studies typically include predominantly male samples, possibly underpowered to detect gender-based differences. In the parent study of the present analysis, careful attention was given to recruiting a gender-balanced sample. Although not without limitations, our findings highlight the necessity to investigate gender-based differences in canine-assisted interventions in veterans, as female and male veterans may present unique healthcare needs and health characteristics [20], warranting tailored interventions. Our results of greater improvements in female (compared to male) participants may be related to additional factors of the intervention and potential underlying mechanisms (which are plausible but were not tested in the present analysis), for example, the veteran-to-veteran altruistic component.

### 4.1. Potential Underlying Mechanisms and Future Research

Our findings of improved psychosocial outcomes and perceived physical health align with research on the association between altruism and well-being benefits. For example, a meta-analysis on the associations between prosociality and well-being found stronger associations in women givers compared to men, and especially with perceived physical health [33]. It is possible that the altruistic component of veterans training service dogs for other veterans had an additional benefit for the female participants, beyond the human–animal bond (with the program or pet dog). Additionally, research on the response of shelter dogs to women compared to men walkers showed that dogs may be more stressed around men and more comfortable around women [24]. Thus, in our sample, beyond the altruistic component and sense of purpose in both groups, female participants in the group who interacted with dogs may have experienced better responses from the dogs (compared to male participants), which in turn contributed to greater improvements in psychosocial outcomes. These potential mechanisms were untested in the present analysis and require future validation in mediation analyses. Nevertheless, a small to moderate effect size was observed in male participants for PTSD symptom reduction, suggesting some effect in the male participants in our sample. As such, it is possible that men would necessitate longer or more frequent bonding time with the dog, or maybe even adopt a dog, to yield similar beneficial outcomes. Still, the difference in the response between the female and male participants may also result from other reasons, such as underlying social and/or individual characteristics which require direct testing.

In our sample, a higher percentage of male (compared to female) participants reported combat exposure. On the other hand, although not assessed in our sample, more female participants endure sexual trauma [3,4]. Thus, it is possible that the history of trauma (e.g., type, childhood, timing) may be related to the effectiveness of the short dog training interventions, with potentially different underlying mechanisms involved. Although not statistically significant, and not prevalent overall, more male participants reported suicidality at baseline than female participants (32.3% and 21.4%, respectively). While our exploratory descriptive analysis suggested that participants with suicidality at baseline reduced their PTSD symptom severity over time, suicidality may be a factor that can hinder the effectiveness of service dog training programs, warranting specific adjunct components to reduce suicidality. Targeted protocols (e.g., motivational interviewing [52]) can serve as an adjunct and effective prerequisite component in dog training programs for veterans with PTSD and suicidality. Future research should strive to understand whether and how the human–animal bond can be utilized to alleviate suicidality.

In summary, different individuals (based on gender or other individual differences) may benefit from more personally tailored interventions. Adjusting modifiable intervention components such as dose (duration and frequency of training sessions), context (group or individual), or canine partner (one or changing dogs) may optimize the response. Such adaptations should be evaluated in rigorously designed and adequately powered studies.

### 4.2. Limitations

The present secondary analysis has several limitations. First, the analysis is based on a small sample, limited power, and multiple testing, with two subgroups analyzed together despite receiving two different interventions. Second, the parent study, with potential selection bias (e.g., highly educated White sample), recruited veterans who self-reported ever being diagnosed with PTSD. Consequently, our sample was heterogeneous and included some participants with managed PTSD symptoms, scoring below the commonly accepted 31 cutoff score on the PCL-5 at baseline. As verified PTSD diagnosis is typically an inclusion criterion, future research should aim to target and characterize veterans who may not meet the PTSD diagnosis criteria, yet suffer from some level of lingering post-traumatic stress symptoms, and may benefit from structured interventions utilizing the human–animal bond. Third, with survey errors regarding the suicidality measure in the present study, future research should also examine the effects of human–animal bond interventions in veterans with PTSD and suicidality (ideation and intent). Lastly, the present analysis is secondary and exploratory in nature. Although this study was not designed to examine differences between female and male participants, our findings raise important issues in the literature, calling for gender-balanced samples and highlighting the need to study and address the unique needs of female or male veterans in general, and specifically in human–animal bond interventions.

## 5. Conclusions

Our secondary analysis of the results of two service dog training programs (a hands-on program and a virtual program) suggests that female participants may respond better than male participants for certain psychosocial outcomes. Future research should aim to replicate the findings with larger and more diverse samples. Furthermore, future research should aim to recruit gender-balanced samples to enable gender-based analyses. Such research will advance the understanding of the unique needs and response of female and male veterans to different animal-based interventions. More targeted research is needed to develop, optimize, and tailor unique and effective interventions to maximize the health benefits of the human–animal bond in veterans with PTSD.

## Figures and Tables

**Table 1:** Participant characteristics for the full sample and gender-based groups.

	Full Sample *N* = 59	Female *n* = 28	Male *n* = 31	*p*
Age, *y*, Median (Q1–Q3)	45 (38–56)	43.5 (38–54.5)	50 (40–57)	*t*_(57)_ = −0.93, *p* = 0.359
Race, *n* (%)				*p* = 0.793
Black/African American	15 (25.4)	8 (28.6)	7 (22.6)	
White	37 (62.7)	17 (60.7)	20 (64.5)	
Other	6 (10.2)	2 (7.1)	4 (12.9)	
Missing	1 (1.7)	1 (3.6)	0 (0.0)	
Ethnicity, *n* (%)				*p* = 1.000
Hispanic/LatinX	6 (10.2)	3 (10.7)	3 (9.7)	
Non-Hispanic/LatinX	52 (88.1)	25 (89.3)	27 (87.1)	
Unknown/not reported	1 (1.7)	0 (0.0)	1 (3.2)	
Highest education, *n* (%)				*p* = 0.301
High school or some college (no degree)	13 (22.0)	3 (10.7)	10 (32.3)	
Associate degree/vocational training	6 (10.2)	3 (10.7)	3 (9.7)	
Bachelor’s degree	11 (18.6)	6 (21.4)	5 (16.1)	
Master’s degree	26 (44.1)	13 (46.4)	13 (41.9)	
Doctorate degree	3 (5.1)	3 (10.7)	0 (0.0)	
Marital status, *n* (%)				χ^2^ = 2.97, *p* = 0.226
Married or domestic partner	34 (57.6)	13 (46.4)	21 (67.7)	
Single, never married	11 (18.6)	6 (21.4)	5 (16.1)	
Previously married	14 (23.7)	9 (32.1)	5 (16.1)	
Pet owner, *n* (%)				*p* = 0.181
Yes	32 (54.2)	16 (57.1)	16 (51.6)	
No	12 (20.3)	3 (10.7)	9 (29.0)	
Missing	15 (25.4)	9 (32.1)	6 (19.4)	
Military branch, *n* (%)				*p* = 1.000
Army	30 (50.8)	14 (50.0)	16 (51.6)	
Air Force	10 (16.9)	7 (25.0)	3 (9.7)	
Navy	7 (11.9)	3 (10.7)	4 (12.9)	
Marine Corps	10 (16.9)	2 (7.1)	8 (25.8)	
Missing	2 (3.4)	2 (7.1)	0 (0.0)	
Combat exposure, *n* (%)	32 (54.2)	10 (35.7)	22 (71.0)	*p* = 0.009
PCL-5 at baseline, *M* (*SD*)	41.34 (17.33)	43.57 (16.73)	39.32 (17.88)	*t*_(57)_ = 0.94, *p* = 0.351
PTSD at baseline, *n* (%)	39 (66.1)	20 (71.4)	19 (61.3)	*p* = 0.582
Suicidality at baseline, *n* (%)	16 (27.1)	6 (21.4)	10 (32.3)	*p* = 0.393

Note. PCL-5 at baseline = mean post-traumatic stress symptoms severity score at baseline, pre-intervention. PTSD = post-traumatic stress disorder. PTSD at baseline = *n* and percentage of participants with a PCL-5 score of 31 or higher at baseline, commonly accepted as PTSD cutoff [35]. Suicidality was determined by the sum score of the screening items (items 4 and 5) of the Beck Suicidality Scale, where any score above 0 refers to some level of suicidality. To test for differences between females and males in categorical variables with small cell sizes, categories were combined. Race was recoded to White/non-White. Ethnicity was recoded to Hispanic/not Hispanic. Highest education was recoded to bachelor’s degree or lower/master’s degree or higher. Pet ownership was recoded to yes/no. Military branch was recoded to Army/other branches. Differences were assessed with a parametric *t*-test for age and PCL-5 at baseline, a chi-square test for marital status, and Fisher’s exact tests for all other variables. Percentages refer to columns (gender-based groups). Percentages may not sum to 100 due to rounding.

**Table 2:** Means and standard deviation of all outcomes for the full sample and female and male participants.

	Full Sample, *M* (*SD*)		Female, *M* (*SD*)		Male, *M* (*SD*)	
Outcome (Range)	Baseline (*n* = 59)	Week 8 (*n* = 51)	Baseline (*n* = 28)	Week 8 (*n* = 23)	Baseline (*n* = 31)	Week 8 (*n* = 28)
PTSD (0–80)	41.34 (17.33)	33.66 (18.87) ^a^	43.57 (16.73)	32.35 (17.69)	39.32 (17.88)	34.78 (20.09) ^a^
Perceived stress (10–50)	31.97 (5.92)	29.94 (7.45)	33.71 (5.32)	29.91 (7.63)	30.39 (6.08)	29.96 (7.45)
Anxiety (8–40)	24.72 (6.94) ^a^	23.14 (8.76)	25.52 (6.62) ^a^	22.78 (8.16)	24.03 (7.24)	23.43 (9.37)
Depression (8–40)	21.31 (7.78)	20.43 (9.62)	21.11 (7.50)	20.09 (9.50)	21.48 (8.14)	20.71 (9.89)
Positive affect (15–75)	42.08 (12.12)	44.35 (14.25)	41.25 (11.21)	45.22 (14.25)	42.84 (13.03)	43.64 (14.47)
Resilience (0–40)	24.59 (6.55) ^a^	25.02 (8.85)	25.68 (5.64)	26.09 (8.27)	23.57 (7.24) ^a^	24.14 (9.36)
Satisfaction with participation in social activities (7–35)	18.31 (7.33)	19.73 (7.82)	17.21 (7.15)	19.65 (7.74)	19.29 (7.47)	19.79 (8.02)
Relationships closeness–avoidance (18–90)	61.76 (10.97)	60.73 (10.42)	64.25 (10.37)	60.91 (10.83)	59.52 (11.17)	60.57 (10.26)
Relationships security–anxiety (16–80)	47.15 (11.41)	44.84 (12.12)	49.54 (12.66)	47.13 (12.19)	45.00 (9.86)	42.96 (11.95)
Satisfaction with social roles (4–20)	12.47 (4.01)	12.98 (4.78)	11.75 (4.60)	12.96 (4.80)	13.13 (3.32)	13.00 (4.85)
Companionship (4–20)	13.84 (3.84) ^a^	14.00 (4.47)	13.64 (3.68)	14.26 (4.34)	14.03 (4.03) ^a^	13.79 (4.65)
Perceived physical health (6–26)	17.12 (5.16)	17.86 (4.84)	16.36 (4.95)	18.43 (5.06)	17.81 (5.33)	17.39 (4.69)
Perceived mental health (6–33)	18.26 (5.26) ^a^	19.73 (6.45)	17.81 (4.65) ^a^	20.04 (6.12)	18.65 (5.79)	19.46 (6.80)

Note. PTSD = post-traumatic stress disorder. Higher scores reflect worse psychosocial conditions for PTSD, perceived stress, anxiety, depression, relationships quality closeness–avoidance, and relationships quality security–anxiety. Higher scores reflect improved (better) conditions for positive affect, resilience, satisfaction with participation in social activities, satisfaction with social roles, companionship, perceived physical health, and perceived mental health. ^a^ number of participants was decreased by one due to incomplete data.

**Table 3:** Results of linear mixed-effects time X gender models and within-group effect sizes in female and male participants per outcome.

Outcome	Estimate	SE	*p*	95% CI	Females Cohen’s *d* (95% CI)	Males Cohen’s *d* (95% CI)
PTSD	7.63	3.67	**0.042**	0.27, 14.99	0.70 (0.24, 1.15)	0.42 (0.03, 0.82)
Perceived stress	3.62	1.45	**0.015**	0.72, 6.52	0.67 (0.21, 1.11)	0.14 (−0.24, 0.51)
Anxiety	2.12	1.51	0.167	−0.91, 5.15	0.52 (0.07, 0.96)	0.13 (−0.24, 0.50)
Depression	0.45	1.55	0.774	−2.66, 3.56	0.28 (−0.14, 0.69)	0.20 (−0.18, 0.57)
Positive affect	−3.36	2.66	0.213	−8.70, 1.99	−0.38 (−0.80, 0.05)	−0.12 (−0.49, 0.25)
Resilience	0.37	1.66	0.826	−2.96, 3.70	−0.10 (−0.51, 0.31)	−0.19 (−0.57, 0.19)
Satisfaction with participation in social activities	−2.07	1.25	0.105	−4.57, 0.44	−0.54 (−0.98, −0.10)	−0.22 (−0.59, 0.16)
Relationships closeness–avoidance	4.26	1.82	**0.023**	0.60, 7.91	0.47 (0.04, 0.90)	−0.17 (−0.55, 0.20)
Relationships security–anxiety	0.86	2.41	0.721	−3.97, 5.69	0.36 (−0.07, 0.78)	0.17 (−0.20, 0.55)
Satisfaction with social roles	−1.04	0.86	0.233	−2.76, 0.69	−0.30 (−0.71, 0.12)	0.03 (−0.34, 0.40)
Companionship	−0.99	0.90	0.278	−2.80, 0.82	−0.26 (−0.67, 0.16)	0.05 (−0.32, 0.42)
Perceived physical health	−1.87	0.79	**0.022**	−3.45, −0.28	−0.61 (−1.05, −0.16)	0.06 (−0.31, 0.43)
Perceived mental health	−1.41	1.21	0.248	−3.84, 1.02	−0.49 (−0.92, −0.05)	−0.24 (−0.62, 0.14)

Note. CI = confidence intervals. PTSD = post-traumatic stress disorder. Significant interactions in bold. *p*-values should be interpreted with caution due to multiple analyses involving correlated variables. Negative Cohen’s *d* values indicate increases in scores compared to baseline, positive scores indicate reductions in scores compared to baseline.

## Data Availability

De-identified data will be available upon request from the corresponding author based on NIH and institutional data sharing policies.

## Supplementary Materials

The following supporting information can be downloaded at: https://www.mdpi.com/article/10.3390/healthcare14091253/s1, Table S1: Results of Time X Group X Gender interaction in linear mixed effect models.
