# Supplementary material for: Service Dog Training Interventions for Veterans with Post-Traumatic Stress: Examining Gender-Based Differences in Psychosocial Outcomes
Source: Healthcare (Basel). 2026 May 6;14(9):1253. doi: 10.3390/healthcare14091253 (PMC13164097; doi:10.3390/healthcare14091253)
Supplement: Supplementary file 1 [file healthcare-14-01253-s001.zip › healthcare-4239328-supplementary.pdf]

Supplemental Material

Service-Dog Training Interventions for Veterans with Post Traumatic Stress: Examining Gender-Based Differences in Psychosocial Outcomes

Shahar Almog, Cheryl A. Krause-Parello, Alejandra Quintero, Deborah Taber & Erika Friedmann

As preparatory analyses before pooling both arms of the parent study, we tested the Time X Group X Gender interaction on the different psychosocial outcomes. The following linear mixed-effect models revealed a non-significant interaction in any of the psychosocial outcomes.

Table S1. Results of Time X Group X Gender interaction in linear mixed effect models.

Outcome	Estimate	SE	*p*	95% CI
PTSD	-1.79	7.34	.808	-16.52, 12.94
Perceived stress	.81	2.91	.782	-5.02, 6.64
Anxiety	-.15	3.06	.961	-6.28, 5.98
Depression	-1.26	3.12	.688	-7.51, 4.99
Positive affect	-6.58	5.30	.220	-17.22, 4.06
Resilience	-2.86	3.32	.394	-9.52, 3.81
Satisfaction with participation in social activities	-1.19	2.52	.640	-6.25, 3.87
Relationships closeness-avoidance	2.41	3.66	.512	-4.92, 9.75
Relationships security-anxiety	2.89	4.73	.544	-6.60, 12.38
Satisfaction with social roles	-1.01	1.73	.563	-4.47, 2.46
Companionship	-.70	1.80	.699	-4.32, 2.91
Perceived physical health	-.82	1.59	.609	-4.00, 2.37
Perceived mental health	-2.98	2.42	.223	-7.83, 1.87
